# Transcervical, intrauterine ultrasound-guided radiofrequency ablation of uterine fibroids with the VizAblate® System: three- and six-month endpoint results from the FAST-EU study

**DOI:** 10.1007/s10397-014-0873-1

**Published:** 2014-11-28

**Authors:** Marlies Bongers, Hans Brölmann, Janesh Gupta, José Gerardo Garza-Leal, David Toub

**Affiliations:** 1 Máxima Medisch Centrum, Veldhoven, The Netherlands; 2 Vrije Universiteit Medisch Centrum, Amsterdam, The Netherlands; 3 Birmingham Women’s Hospital, Birmingham, UK; 4 Universidad Autónoma de Nuevo León, Monterrey, Nuevo Leon Mexico; 5 Gynesonics, Inc., Redwood City, CA USA; 6 Department of Obstetrics and Gynecology, Albert Einstein Medical Center, Philadelphia, PA USA

**Keywords:** Fibroids, Radiofrequency ablation, VizAblate, Intrauterine sonography

## Abstract

This was a prospective, longitudinal, multicenter, single-arm controlled trial, using independent core laboratory validation of MRI results, to establish the effectiveness and confirm the safety of the VizAblate® System in the treatment of symptomatic uterine fibroids. The VizAblate System is a transcervical device that ablates fibroids with radiofrequency energy, guided by a built-in intrauterine ultrasound probe. Fifty consecutive women with symptomatic uterine fibroids received treatment with the VizAblate System. Patients had a minimum Menstrual Pictogram score of 120, no desire for fertility, and met additional inclusion and exclusion criteria. The VizAblate System was inserted transcervically and individual fibroids were ablated with radiofrequency energy. An integrated intrauterine ultrasound probe was used for fibroid imaging and targeting. Anesthesia was at the discretion of each investigator. The primary study endpoint was the percentage change in perfused fibroid volume, as assessed by contrast-enhanced MRI at 3 months. Secondary endpoints, reached at 6 months, included safety, percentage reductions in the Menstrual Pictogram (MP) score and the Symptom Severity Score (SSS) subscale of the Uterine Fibroid Symptom-Quality of Life questionnaire (UFS-QOL), along with the rate of surgical reintervention for abnormal uterine bleeding and the mean number of days to return to normal activity. Additional assessments included the Health-Related Quality of Life (HRQOL) subscale of the UFS-QOL, medical reintervention for abnormal uterine bleeding, and procedure times. Fifty patients were treated, representing 92 fibroids. Perfused fibroid volumes were reduced at 3 months by an average of 68.8 ± 27.8 % (*P* < 0.0001; Wilcoxon signed-rank test). At 6 months, mean MP and SSS scores decreased by 60.8 ± 38.2 and 59.7 ± 30.4 %, respectively; the mean HRQOL score increased by 263 ± 468 %. There were two serious adverse events (overnight admissions for abdominal pain and bradycardia, respectively) and no surgical reinterventions. These 6-month results suggest that the VizAblate System is safe and effective in providing relief of abnormal uterine bleeding associated with fibroids, with appropriate safety and a low reintervention rate.

## Introduction

Uterine fibroids are the most prevalent benign uterine tumors and have an age-specific cumulative incidence in the United States that is nearly 70 % among white women and greater than 80 % among black women [1]. Despite the availability of several alternatives to hysterectomy, over 200,000 hysterectomies are performed annually for fibroids in the United States [1, 2]. More than 150 years after the first abdominal hysterectomy for fibroids, there remains a lack of consensus regarding what constitutes the “gold standard” treatment for fibroids against which all other treatment options may be compared [3]. Nonetheless, effective minimally invasive alternatives to hysterectomy exist to accommodate the growing preference of many women for uterine preservation.

Radiofrequency ablation (RFA) has been used as a fibroid treatment modality since the early 1990s, with multiple clinical studies confirming its safety and efficacy [4–10]. It has been shown that radiofrequency ablation produces thermal fixation and coagulative necrosis within the treated fibroids [9, 11]. Recent studies have been performed using RFA in conjunction with simultaneous, real-time sonography to enable volumetric ablations, resulting in volume reduction and symptom improvement [8, 9, 12].

The VizAblate System® (Gynesonics, Redwood City, CA) is a new medical device that has received CE Mark for distribution in the European Union. It combines radiofrequency ablation for the transcervical treatment of uterine fibroids with intrauterine sonography for real-time imaging [13]. The Fibroid Ablation Study-EU (FAST-EU) is a study designed to examine the safety and effectiveness of transcervical radiofrequency ablation of uterine fibroids under intrauterine sonography guidance with the VizAblate System. This report presents the 6-month endpoint results of 50 women treated under the FAST-EU study and is the first report of patient efficacy data after transcervical radiofrequency ablation of fibroids under intrauterine sonography guidance.

## Materials and methods

### Patient selection

This study is a prospective, non-randomized, single-arm, multicenter controlled trial using independent core laboratory validation of MRI results. Patients were enrolled from a total of seven sites in three nations: Mexico (one site), The United Kingdom (two sites), and The Netherlands (four sites). The protocol was approved by the Ethics Committees of the respective institutions as well as by the Federal Commission for Protection against Health Risks (COFEPRIS) in Mexico. All enrolled patients provided written informed consent for treatment with the VizAblate System prior to enrollment. The study overview was published on ClinicalTrials.gov (identifier—NCT01226290) and conducted in accordance with Standard ISO 14155 (Clinical investigation of medical devices for human subjects—Good clinical practice) of the International Organization for Standardization (ISO), the Helsinki Declaration of 1975, as revised in 2008, and in accordance with the ethical standards of applicable national regulations and institutional research policies and procedures governing human experimentation.

Women were eligible for inclusion if they were 28 years of age or older and not pregnant, with regular predictable menstrual cycles and abnormal uterine bleeding for at least 3 months associated with one to five uterine fibroids measuring between 1 and 5 cm in maximum diameter. Fibroids were counted in this total if they had an edge within the inner half of the myometrium; these were termed “target fibroids,” as they are believed to be more likely associated with heavy menstrual bleeding than myomata that are distant from the endometrial cavity. Target fibroids, which therefore were the only fibroids to be ablated, consisted of fibroids of FIGO types 1, 2, 3, 4, and 2–5 (“transmural”). At least one fibroid was required to indent the endometrium, as determined via hysterosonography and/or hysteroscopy and corroborated via contrast-enhanced MRI. All MRI studies were forwarded to a core laboratory (MedQIA, 924 Westwood Blvd., Suite 650, Los Angeles, CA 90024, USA) for quality control and interpretation to reduce variability in the measurements; the core laboratory also developed standardized imaging protocols for use at the individual study sites, credentialed the sites, and trained MRI technologists at each study site. A Menstrual Pictogram score ≥120 was required for inclusion along with a baseline UFS-QOL SSS score ≥20. The Menstrual Pictogram is a variant of the Pictorial Blood Loss Assessment Chart (PBAC) that patients complete to provide a visual assessment of menstrual blood loss during a single cycle [14, 15]. Unlike the original PBAC described by Higham and colleagues, the Menstrual Pictogram includes a greater range of icons representing different saturations of sanitary products, clots and losses in a toilet, and also distinguishes different absorbency levels of sanitary napkins and tampons [16].

Patients were willing to maintain the use or non-use of hormonal contraception from 3 months prior to the study through the 12-month follow-up period. Exclusions included a desire for future fertility, the presence of one or more type 0 myomata, cervical dysplasia, endometrial hyperplasia, active pelvic infection, clinically significant adenomyosis (>10 % of the junctional zone measuring more than 10 mm in thickness as measured by MRI), and the presence of one or more treatable fibroids that were significantly calcified (defined as <75 % fibroid enhancement by volume on contrast-enhanced MRI). Each patient underwent screening that included transvaginal sonography, hysteroscopy or hysterosonography, contrast-enhanced MRI, endometrial biopsy, and a pregnancy test.

Patients were assigned a unique identification number at the time that they provided informed consent for study screening. Patients were considered “enrolled” in the study once adherence with all inclusion and exclusion criteria had been verified and documented. All records were de-identified and only the range of each patient’s age was documented, as per clinical trial requirements in The Netherlands. Women were followed at 7–14 days, 30 days, 3 months, 6 months, and at 12 months post-treatment.

The primary study endpoint was the percentage change in target fibroid perfused volume as assessed by contrast-enhanced MRI at baseline and again at 3 months. The patient success criterion was >30 % reduction in mean target fibroid perfused volume at 3 months in at least 50 % of patients.

Additional endpoints, reached at 6 months, included safety, percentage reductions in the Menstrual Pictogram (MP) score and the Symptom Severity Score (SSS) subscale of the Uterine Fibroid Symptom-Quality of Life questionnaire (UFS-QOL), the rate of surgical reintervention for abnormal uterine bleeding, and the mean number of days to return to normal activity. While there is no absolute level below which the SSS would be considered as “within normal limits,” a population of 29 healthy women without uterine fibroids was demonstrated by Spies and colleagues to have an average SSS of 22.5 ± 22.1 [17]. Additional assessments included the Health-Related Quality of Life (HRQOL) subscale of the UFS-QOL, medical reintervention for menorrhagia, and procedure times (as recorded from the start of transvaginal sonography to the end of RF ablation). Lower scores on the SSS subscale are desirable; conversely, higher scores on the HRQOL subscale are preferable. For the SSS, a 10-point reduction in the score is generally considered clinically significant [17–19].

### Statistical analysis

The null hypothesis for this study endpoint is H_0_: probability of success <50 % versus the alternative H_a_: probability of success ≥50 %. Thus, the lower bound of the two-sided 95 % confidence interval on the observed probability of success must be greater than or equal to 50 %. We anticipated that at least 90 % of the patients would remain in the set of patients who were not excluded from analysis, and that the true probability of success for included patients would be 72.0 %. If this is the case, a sample of 40 patients is sufficient to detect this difference of 22 % in probability of success with a power of 82 % using a one-group chi-square test with a 0.05 two-sided significance level. Allowing for an expected dropout rate of 20 % at the 12-month follow-up visit, the minimum recommended sample size for the initial study protocol was 48. The primary study endpoint success criterion was achievement of >30 % reduction in mean target fibroid perfused volume in at least 50 % of patients. The primary endpoint analysis is performed on a per-fibroid basis, rather than using a subjective “dominant fibroid” in a given patient who could have multiple similar fibroids that were ablated.

The Full Analysis dataset includes all patients enrolled under the protocol who provided a baseline fibroid volume assessment and received treatment with the VizAblate System. The Per-Protocol dataset includes all patients enrolled under the protocol who received treatment with the VizAblate System and provided both a baseline fibroid volume assessment and a 3-month assessment and/or who received a surgical reintervention. Patients who received a surgical reintervention were considered treatment failures. Any patients with major protocol deviations that were considered to influence a treatment evaluation were excluded (e.g., pregnancy, concomitant procedures, medical reintervention). The Per-Protocol dataset was used as the primary analysis set for the primary endpoint analysis.

All statistical analyses were performed with SAS 9.3 (SAS, Cary, NC). Values were considered significant at the level of α = 0.05. The Wilcoxon signed-rank test was used to test if a change was significantly different from 0. Missing data were not imputed for patients included in this Per-Protocol analysis.

### Procedure

**Fig. 1:**
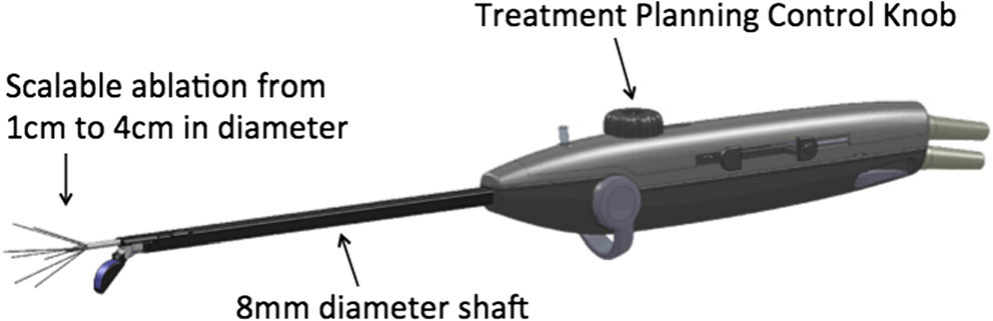
The VizAblate treatment device

**Fig. 2:**
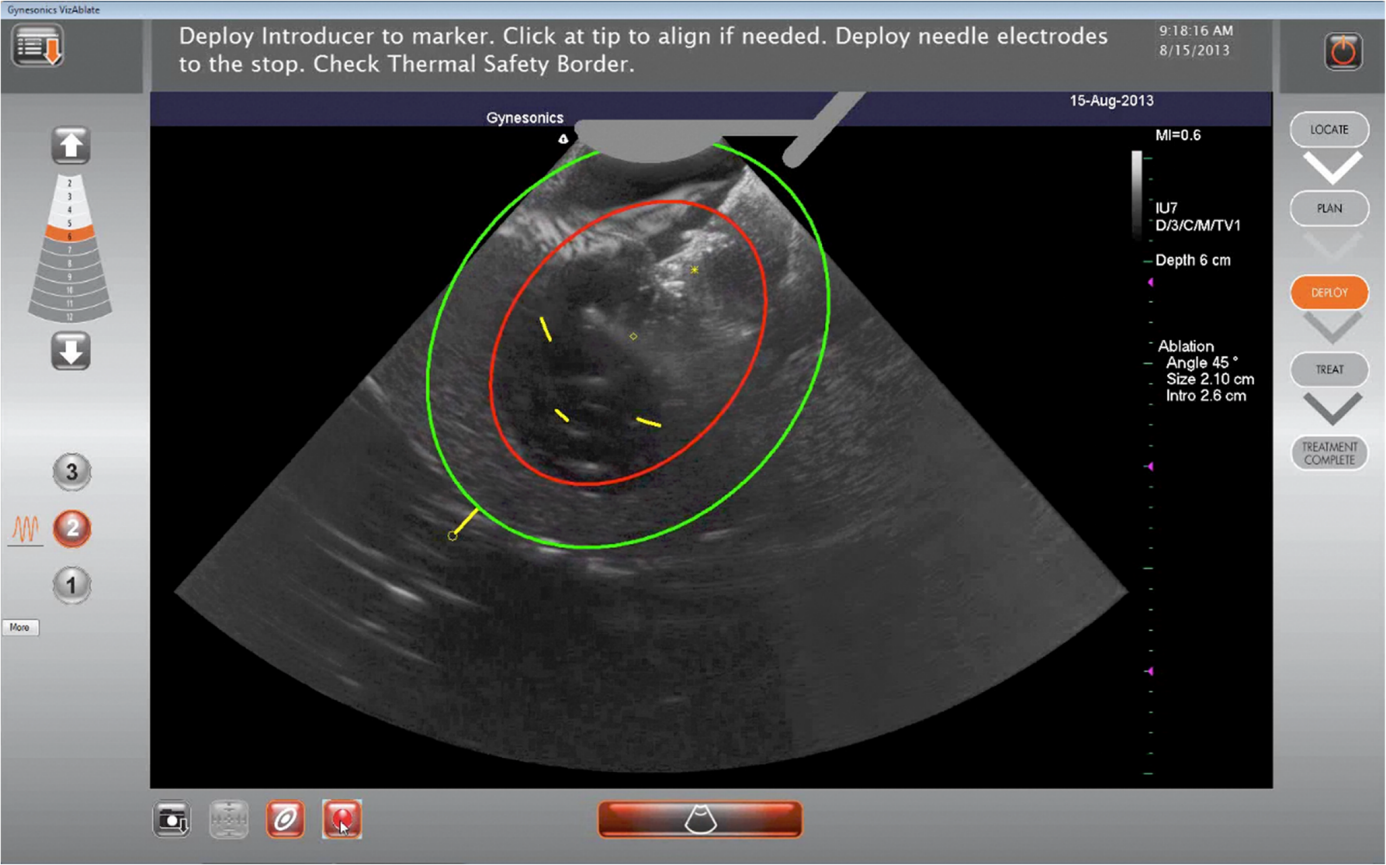
Intrauterine sonogram of a submucosal fibroid with the VizAblate ablation guides visible (Ablation Zone in *red* and Thermal Safety Border in *green*). The Ablation Zone is a two-dimensional representation of the average region of tissue ablation for a selected treatment size. Tissue outside the Thermal Safety Border is at a safe distance from the Ablation Zone and will be preserved. The serosa is visible as an echogenic border around the uterus

The VizAblate System consists of a reusable intrauterine ultrasound (IUUS) probe and a single-use, disposable articulating RF handpiece that are combined into a single treatment device (Fig. 1). Other integrated components of the VizAblate System include an RF generator and an ultrasound system with a custom graphical user interface. This graphical user interface provides the gynecologist with an image-guided treatment system that indicates the borders of the thermal ablation as well as the border beyond which thermal heating is not present (Fig. 2).

Pregnancy was excluded before the procedure by utilizing a urine pregnancy test. Anesthesia was chosen by each investigator in consultation with an anesthesiologist; options included general inhalational anesthesia, regional anesthesia, and conscious sedation with or without paracervical blockade. Two dispersive electrode pads were placed, one on each anterior thigh; the dispersive electrodes contain thermocouples positioned at each leading edge for skin temperature monitoring. Just before insertion of the VizAblate Handpiece, transvaginal sonography was employed at the discretion of each investigator to confirm the presence, location, and size of each fibroid.

Transvaginal sonography, if desired for fibroid mapping before insertion of the VizAblate Handpiece, was performed with a transvaginal ultrasound probe as provided with the VizAblate System. After achieving cervical dilatation to 8 mm, the integrated VizAblate Handpiece (containing the RF electrode array and the intrauterine ultrasound probe) was inserted transcervically into the uterus. A small volume (generally 10–15 mL) of hypotonic fluid such as sterile water or 1.5 % glycine was infused through the device for acoustic coupling. Leiomyomata were then visualized with IUUS and mapped in a systematic fashion within the uterus.

After articulating the ultrasound probe, the investigators used the graphical overlay to simulate various ablation widths, angles, and locations of the intended ablation. In this fashion, before introducing the RF electrodes into a fibroid, the investigator planned and optimized the ablation. Once the size, angle, and location of the ablation were established, the investigator advanced a trocar-tipped introducer into the fibroid under intrauterine ultrasound visualization. The investigator aligned the graphical overlay with the introducer tip and then rotated the VizAblate Handpiece about the introducer to assess the position of the Ablation Zone and Thermal Safety Border relative to the uterine serosa, as displayed on the graphical user interface, adjusting the size and/or position of the desired ablation if necessary. Depending on the width of the ablation, the distance from the Ablation Zone to the uterine serosal margin will vary from 6.0 to 9.5 mm. When properly positioned, the introducer is located within the fibroid while the serosa is maintained tangent to or beyond the Thermal Safety Border. The investigator then deployed the electrodes, again rotating to ascertain the position of the Thermal Safety Border relative to the serosa. Once the Thermal Safety Border was confirmed to be within the uterine serosal margin in all adjacent ultrasound planes, RF energy was used to accomplish the ablation. Treated fibroids each received one or more ellipsoidal ablations, ranging from 1 to 4 cm in width and 2–5 cm in length. The number of ablations, along with their sizes, was at the discretion of the investigator. While usually only one ablation may sufficiently ablate up to a 5-cm myoma, two smaller ablations might be needed rather than one larger one, in order to ablate a greater aggregate volume when a fibroid is close to the serosa or where there is additional constraint on ablation volume and position (e.g., the cornu).

The VizAblate System delivers up to 150 W of RF energy for a preset duration. The RF generator controls energy delivery to maintain a constant temperature of 105 °C at the needle electrodes. Upon completion of RF treatment, the needle electrodes were retracted along with the introducer, the ultrasound articulation angle was reset to 0°, and the device was withdrawn.

Throughout this study, the investigator was asked to treat fibroids to maximize the ablated volume of each treated fibroid while maintaining the thermal safety border within the uterine serosal margin. In doing so, the investigator determined the best ablation size and location for each individual fibroid, and whether one or more ablations should be performed. The intent was to ablate all target fibroids (up to the eligibility limit of 5) present within a given patient. At each center, only a single physician would perform each procedure in the interests of consistency and quality.

## Results

### Patients

Fifty patients were treated in the FAST-EU study at seven sites. On average, each site treated 7.1 patients ±7.8 (median 5.0; range 1–23), with four centers treating five or more patients and the remaining three treating one to two patients. Baseline characteristics for all treated study patients are provided in Table 1. Anesthesia was provided as noted in Table 2. The average procedure time (sonography time plus Treatment Device time) was 38.8 ± 22.5 min (range, 11–95.5 min).

**Table 1:** Baseline patient characteristics

Subjects treated	50
Most frequent age range	41–45 years^a^
Mean menstrual pictogram (MP) score	423 ± 253 (range, 119–1582)
Mean UFS-QOL SSS score	61.7 ± 16.9 (range, 28.1–100.0)
Mean UFS-QOL HRQOL score	34.3 ± 19.0 (range, 0.0–73.3)
Total number of target fibroids identified on MRI	118
Mean number of target fibroids per patient	2.4 ± 1.7 (range, 1–7)^b^
Mean diameter of target fibroids	2.9 ± 1.4 cm (range, 1.0–6.9 cm)
Mean perfused fibroid volume	18.3 ± 20.6 cc (range, 0.3–77.0 cc)
Mean total (perfused + nonperfused) fibroid volume	18.8 ± 21.4 cc (range, 0.3–77.0 cc)

^a^Subject ages were specified as a range by each site to protect subject privacy

^b^Two small additional fibroids, beyond the upper limit of five target fibroids/patient, were identified on review of one MRI series after treatment

**Table 2:** Anesthesia provided to FAST-EU patients

Anesthesia option	Number of subjects
General anesthesia alone	15 (30.0 %)
Conscious sedation alone	15 (30.0 %)
Spinal anesthesia alone	8 (16.0 %)
Conscious sedation + epidural anesthesia	8 (16.0 %)
Epidural anesthesia alone	2 (4.0 %)
Paracervical blockade alone	1 (2.0 %)
General anesthesia + epidural anesthesia	1 (2.0 %)

### Exclusions from analysis

Two patients (four fibroids) were excluded from a Per-Protocol analysis of the primary endpoint, perfused fibroid volume. These two patients were deemed by the core MRI laboratory to have had unusable imaging for making precise fibroid measurements at the screening (one patient) and/or 3-month (both patients) MRI studies. Good faith efforts were made to obtain MRI studies for these patients that would meet the strict quality control requirements of the core MRI lab (MedQIA). As in the end, accurate fibroid contouring measurements could not be obtained in these two patients with any reasonable degree of reliability; their MRI data did not contribute to the data analysis. There were six (8 %) patients excluded from one or more of the patient-reported outcomes (MP, UFS-QOL). Two patients (4 %) underwent an ancillary fibroid or polyp resection at the time of RF ablation, and as a result, all of their patient-reported outcomes were excluded from a Per-Protocol analysis, including their baseline MP and UFS-QOL results. Three patients (6 %) underwent medical reintervention (e.g., tranexamic acid) for abnormal uterine bleeding within 6 months of the ablation procedure, resulting in exclusion of their patient-reported outcomes at 6 months. One patient reported a pregnancy at the time of her 6-month follow-up visit and was thus excluded from the 6-month analysis. One patient did not turn in her menstrual pictogram or complete the HRQOL portion of the UFS-QOL. Thus, at 6 months there were 43 patients who were included in the analysis of MP and UFS-QOL HRQOL and 44 who had data included in the UFS-QOL SSS analysis.

### Mean percentage reduction in perfused and total fibroid volume

Characteristics of fibroids that were ablated are shown in Table 3 and ablation results at 3 months for both Per-Protocol and Full Analysis datasets are provided in Table 4. Fibroids are classified in Table 3 as per the International Federation of Gynecology and Obstetrics classification system [20]. While the Per-Protocol dataset is intended as the primary analysis source for the primary endpoint, the Full Analysis dataset is included for comparison. As noted in the ablation results, radiofrequency ablation with the VizAblate System was associated with statistically significant reductions in both total and perfused fibroid volumes at 3 months. This was true for both the Full Analysis dataset and Per-Protocol dataset. On a Per-Protocol basis, half of the fibroids experienced at least a 77.1 % reduction in perfused volume and at least a 63.1 % reduction in total volume (76.9 % and 62.5 %, respectively, for the Full Analysis dataset).

**Table 3:** Characteristics of ablated fibroids

Total number of ablated target fibroids^a^	92
Mean number of ablated target fibroids per subject	1.8 ± 1.1 (range, 1–5)
Total number of type 0 ablated fibroids	0
Total number of type 1 ablated fibroids	14
Total number of type 2 ablated fibroids	42
Total number of type 3 ablated fibroids	3
Total number of type 4 ablated fibroids	25
Total number of type 2–5 (transmural) ablated fibroids	8
Mean diameter of ablated fibroids	3.2 ± 1.4 cm (range, 1.1–6.9 cm)

^a^Includes three fibroids that were ablated in a subject whose MRI data was not evaluable with regard to precise fibroid measurements

**Table 4:** Reduction in mean perfused and total fibroid volumes at 3 months (Per-Protocol and Full Analysis datasets)

	Per-Protocol				Full Analysis set			
	Baseline	3 Months	% Reduction from baseline	*P* value^a^	Baseline	3 Months	% Reduction from baseline	*P* value^a^
Number of MRI-evaluable fibroids	89	88			89	89		
Number of subjects	49	48			49	49		
Perfused fibroid volume (cc)	18.3 ± 20.6 9.5 (0.3–77.0)	5.5 ± 9.2 1.6 (0.0–45.7)	68.8 ± 27.8 % 77.1 % (−33.3–100 %)	<0.0001	18.3 ± 20.6 9.5 (0.3–77.0)	5.8 ± 9.6 1.6 (0.0–45.7)	68.1 ± 28.6 % 76.9 % (−33.3–100 %)	<0.0001
Total fibroid volume (cc)	18.8 ± 21.4 9.5 (0.3–77.0)	7.7 ± 11.8 1.9 (0.0–56.3)	55.3 ± 37.2 % 63.1 % (−85.7–100 %)	<0.0001	18.8 ± 21.4 9.5 (0.3–77.0)	8.0 ± 12.0 1.9 (0.0–56.3)	54.7 ± 37.4 % 62.5 % (−85.7–100 %)	<0.0001

Data are mean ± standard deviation; median; (range)

^a^Wilcoxon signed-rank test, null hypothesis of no change

Seventy-nine of 88 treated fibroids (89.8 %), in 48 patients for which perfusion data was available at 3 months post-treatment, met or exceeded the primary study endpoint success criterion (achievement of >30 % reduction in mean target fibroid perfused volume in at least 50 % of patients).

## Patient-reported outcomes

Patient-reported secondary endpoint data are provided in Table 5. A 50 % or greater reduction in MP was achieved in 33 of 43 patients (76.7 %) at 6 months. The median reduction in MP score at 6 months was 70.8 %. Reductions in the MP and SSS subscale of the UFS-QOL are desired, as are increases in the HRQOL subscale of the UFS-QOL.

**Table 5:** Improvement in patient reported outcomes at 6 months

	Baseline	6 Months	% Improvement from baseline	*P* value^a^
MP	48	43	43	<0.0001
	418 ± 251	146 ± 144	60.8 ± 38.2 %	
	361	98	70.8 %	
	(119–1582)	(0–786)	(−73.1–100 %)	
UFS-QOL SSS	48	44	44	<0.0001
	62.1 ± 16.8	23.5 ± 17.8	59.7 ± 30.4 %	
	60.9	18.8	66.7 %	
	(28.1–100)	(0.0–78.1)	(−22.2–100 %)	
UFS-QOL HRQOL	47	43	43	<0.0001
	34.5 ± 18.7	82.5 ± 17.8	263 ± 468 %	
	30.2	86.2	126.0 %	
	(0.0–73.3)	(12.9–100)	(−28.6–2800 %)	

Data are number of subjects; mean ± standard deviation; median; (range)

*MP* menstrual pictogram, *UFS*-*QOL SSS* uterine fibroid symptom and health-related quality of life symptom severity score subscale, *UFS*-*QOL HRQOL* uterine fibroid symptom and health-related quality of life health-related quality of life subscale

^a^Wilcoxon signed-rank test, null hypothesis of no change

As shown in Table 5, the reduction in the transformed SSS subscale of the UFS-QOL questionnaire at 6 months was statistically significant, as was the increase in the transformed HRQOL subscale. Patients experienced nearly a 60 % reduction in SSS at 6 months.

## Device safety

All procedures were successfully completed. The adverse events attributable to the device or procedure consisted of dysmenorrhea (six patients), pelvic cramping (four patients), and abnormal uterine bleeding above the patient’s baseline (seven patients). The abnormal uterine bleeding above baseline was seen in six of the seven patients at time points from 3 to 5 months post-ablation and were felt to have been consistent with fibroid sloughing based on their 3-month MRI studies and clinical presentation. At 6 months, there was a single bulk expulsion involving a 5.4-cm type 1 myoma at 12 days post-ablation. The patient did not initially mention it to her treating physician, noting only bulk passage of tissue (as opposed to gradual sloughing) in response to a physician query after the fibroid had been noted to have essentially disappeared on the 3-month MRI study. Given that it is unlikely that expulsion of a large fibroid could have been largely asymptomatic, presumably much of the fibroid had gradually sloughed off to the point where only a small portion was actually expelled.

There were two readmissions within 30 days of the procedure. One patient was admitted overnight on post-procedure day 9 to receive parenteral antibiotics for a urinary tract infection and was discharged on the following day. Another patient, who had no known history of cardiovascular disease or other medical disorders, developed bradycardia down to 38 bpm shortly after the procedure and was kept overnight in the hospital for treatment with atropine and observation; the patient was discharged the next morning in stable condition.

## Reintervention

No patient underwent surgical intervention within 6 months of treatment. Three patients received medical reintervention with tranexamic acid after presenting at 3 months post-ablation complaining of persistent or increased abnormal uterine bleeding that was felt to have been secondary to fibroid sloughing based on the clinical presentation and 3-month MRI findings of markedly reduced submucous fibroid volume with partial extrusion into the endometrial cavity.

## Pregnancy

There was a single pregnancy reported within the first 6 months after ablation with the VizAblate System. The patient presented with amenorrhea and a positive pregnancy test at her 6-month study visit and delivered a liveborn male infant at term, weighing 3150 g and with Apgar scores of 9 and 9, via elective repeat Cesarean section [21].

## Return to normal activity

A total of 47 patients completed a recovery diary relating to how long it took them to return to their normal activities of daily life. On average, return to normal activity took 4.4 ± 3.1 days (median 4.0 days; range 1–14 days).

## Discussion

Since the 1990s, there have emerged several modalities to treat uterine fibroids, including uterine artery embolization (UAE) and magnetic resonance-guided focused ultrasound (MRgFUS). Uterine artery embolization is a treatment option, with a 20–28.4 % failure rate after 5 years [22, 23]. Of note, UAE is not considered generally applicable for women who desire future pregnancy, and has been associated with post-embolization syndrome and, occasionally in older women, premature ovarian failure [23–28]. Magnetic resonance-guided focused ultrasound, like radiofrequency ablation methods, ablates fibroids using energy to generate hyperthermic tissue temperatures. However, this modality is not widely available and requires up to 3 h per treatment.

The VizAblate System uses an incisionless, transcervical approach to treat symptomatic fibroids, including those that are not generally amenable to alternative intrauterine treatment approaches such as hysteroscopic resection or morcellation. Fibroid ablation takes place under real-time visualization provided by an intrauterine sonography probe that is integrated within the device. This built-in imaging removes the need for the physician to coordinate more than one device and more than one monitor. The graphical user interface delineates the boundaries of ablation and thermal spread outside the zone of ablation and enables the operator to avoid thermal injury to the serosa with its potential for adhesiogenesis and injury to adjacent viscera. Intrauterine sonography provides higher-resolution imaging than the more prevalent transvaginal sonography and has greater precision and accuracy for the measurement of fibroids near the endometrial cavity [29]. The use of intrauterine sonography has been demonstrated useful for the imaging of intratubal implants for sterilization after placement [30].

These 6-month results demonstrate that transcervical intrauterine sonography-guided radiofrequency ablation of fibroids can result in significant reductions in perfused fibroid volumes and fibroid-associated symptoms. In contrast with fibroid treatment modalities that often require 1–3 h to accomplish, mean treatment times with the VizAblate System were less than 40 min. Treatment did not require general anesthesia in nearly 70 % of patients and recovery time was relatively brief. The median time for the return to normal activity was 4.0 days, with a mean of 4.4 ± 3.1 days (range, 1–14 days). This contrasts with laparoscopic radiofrequency ablation of fibroids, in which the largest published study to date reported a median time for returning to normal activities of 9 days (range, 2–60 days) [31].

Transcervical radiofrequency ablation avoids many of the potential complications associated with a laparoscopic or open procedure for the treatment of fibroids. There are no incisions and the uterine serosa is not penetrated or coagulated. There is no entry into the peritoneal cavity, so that intraperitoneal adhesiogenesis is unlikely. There is no overt risk of ureteral injury. In contrast to operative hysteroscopy, only a small quantity of hypotonic fluid is used for acoustic coupling, no large venous sinuses are exposed, and intrauterine pressure is not raised to levels above mean arterial pressure, avoiding significant fluid intravasation. The integral intrauterine sonography probe permits visualization of the myometrium and serosa, permitting a perspective of the myometrium and intramyometrial pathology that are not readily achievable with a hysteroscope.

At 6 months, there have been significant reductions in perfused and total fibroid volumes. Contrast-enhanced MRI demonstrated a significant reduction in the volume of perfused fibroid tissue at 3 months. The 3-month time point was selected in order to provide early data on the treatment impact on fibroid perfusion; it was not assumed prospectively that there would be a significant impact on total fibroid volume. It was suspected that reductions in total fibroid volume, caused by the process of coagulative necrosis, would require a longer time horizon to be demonstrated on contrast-enhanced MRI. As with MRgFUS, which is another form of hyperthermic ablation of uterine fibroids, it is known that sustained symptom relief may be accomplished by ablating as much of the fibroid volume as possible, although it is not necessary to ablate 90 % or more of the fibroid to reduce fibroid symptoms [32]. Nonetheless, there was an average 55.3 % reduction in total fibroid volume at 3 months, with some fibroids achieving 90–100 % reduction in total volume.

At 6 months, there were statistically significant reductions in menstrual blood loss, as evidenced by reductions in menstrual pictogram scores, as well as significant improvements in both subscales of the UFS-QOL questionnaire. No patients underwent surgical reintervention within 6 months. Based on the MP score, bleeding reduction was experienced by 76.7 % of patients at 6 months. It should be noted that Lukes and colleagues defined a reduction in menstrual blood loss of ≥36 cc, or a 22 %, reduction, to be the minimum level of improvement in bleeding that is meaningful to women [33, 34]. Thus, the majority of patients exceeded both standards.

In addition to clinical efficacy, device safety was also acceptable. No patient required emergency surgery and there were no unscheduled returns to the operating room. Adverse events were generally minor and anticipated.

Seven patients experienced abnormal uterine bleeding above their baseline levels, generally around 3 months post-treatment. This was felt to be secondary to fibroid sloughing; MRI studies at 3 months typically revealed extrusion of ablated fibroids into the endometrial cavities with loss of total fibroid volume. As fibroids undergo coagulative necrosis after radiofrequency ablation, some patients may experience sloughing phenomena, particularly if the fibroid is already partially within the endometrial cavity or, as we speculate, the fibroid pseudocapsule has been ablated. As all patients are required to have at least one submucosal fibroid for entrance into the study, it is perhaps not surprising that 6 of 50 patients (12 %) had presumptive fibroid sloughing secondary to radiofrequency ablation. Consistent with reports of patient experiences after MRgFUS, gross expulsion of ablated fibroids was unusual, occurring in only one patient. Fibroid expulsion is more common after uterine artery embolization, in contrast to the experience of thermal ablation methods like MRgFUS and RF ablation [35–37].

Of the seven centers that treated patients in the FAST-EU study, half of the centers treated at least five patients. There was no unusual learning curve; the procedure time varied with the complexity of the case, with some first cases taking 14.5–18 min and others involving four to five larger fibroids requiring much longer.

This study has several advantages. Care was taken to exclude women with suspected anovulation through a strict inclusion criterion regarding the menstrual history, along with requiring the presence of at least one fibroid that indented the endometrial cavity. The latter requirement makes it more likely that a patient’s bleeding symptoms are secondary to fibroids rather than another etiology. A core MRI facility was used to reduce variability and bias in MRI imaging quality, interpretation, and measurements relative to the primary study endpoint. In addition, the use of multiple clinical sites that include academic medical centers as well as community hospitals provides a more realistic assessment of the use of the VizAblate System than would a single site.

Nonetheless, there are some limitations to this study. The study is a nonrandomized single-arm study and does not include a sham or another fibroid treatment for comparison. It was not intended to examine outcomes for women with fibroids significantly larger than 5.0 cm, with the largest fibroid being 6.9 cm in diameter; additional study will be required to examine ablation outcomes in women with larger fibroid volumes. Finally, this report includes outcomes through the first 6 months after treatment with the VizAblate System; longer-term and comparative data are needed, are being acquired, and will be reported separately.

## Conclusions

Based on the 6-month data from the FAST-EU study, radiofrequency ablation of uterine fibroids is an effective treatment modality that preserves the uterus. When performed transcervically with the VizAblate System, the integrity of the uterine serosa and abdominal cavity are not violated, concurrent intrauterine sonography provides a straightforward method for targeting myomata associated with abnormal uterine bleeding, and general anesthesia is not required.
